# Towards the future exploration of mucosal mRNA vaccines against emerging viral diseases; lessons from existing next-generation mucosal vaccine strategies

**DOI:** 10.1038/s41541-022-00485-x

**Published:** 2022-06-28

**Authors:** Sodiq A. Hameed, Stephane Paul, Giann Kerwin Y. Dellosa, Dolores Jaraquemada, Muhammad Bashir Bello

**Affiliations:** 1 Univ Lyon, Université Claude Bernard Lyon 1, 69100 Villeurbanne, France; 2 CIRI – Centre International de Recherche en Infectiologie, Team GIMAP, Univ Lyon, Université Claude Bernard Lyon 1, Inserm, U1111, CNRS, UMR530, CIC 1408 Vaccinology, F42023 Saint-Etienne, France; 3 Universidad Autónoma de Barcelona, 08193 Cerdanyola, Spain; 4 Department of Veterinary Microbiology, Faculty of Veterinary Medicine, Usmanu Danfodiyo University PMB, 2346 Sokoto, Nigeria

**Keywords:** RNA vaccines, RNA vaccines, Viral infection

## Abstract

The mRNA vaccine platform has offered the greatest potential in fighting the COVID-19 pandemic owing to rapid development, effectiveness, and scalability to meet the global demand. There are many other mRNA vaccines currently being developed against different emerging viral diseases. As with the current COVID-19 vaccines, these mRNA-based vaccine candidates are being developed for parenteral administration *via* injections. However, most of the emerging viruses colonize the mucosal surfaces prior to systemic infection making it very crucial to target mucosal immunity. Although parenterally administered vaccines would induce a robust systemic immunity, they often provoke a weak mucosal immunity which may not be effective in preventing mucosal infection. In contrast, mucosal administration potentially offers the dual benefit of inducing potent mucosal and systemic immunity which would be more effective in offering protection against mucosal viral infection. There are however many challenges posed by the mucosal environment which impede successful mucosal vaccination. The development of an effective delivery system remains a major challenge to the successful exploitation of mucosal mRNA vaccination. Nonetheless, a number of delivery vehicles have been experimentally harnessed with different degrees of success in the mucosal delivery of mRNA vaccines. In this review, we provide a comprehensive overview of mRNA vaccines and summarise their application in the fight against emerging viral diseases with particular emphasis on COVID-19 mRNA platforms. Furthermore, we discuss the prospects and challenges of mucosal administration of mRNA-based vaccines, and we explore the existing experimental studies on mucosal mRNA vaccine delivery.

## Introduction

Since time immemorial, emerging viral diseases have been threats to humanity all over the world^1^. They are responsible for the death of millions of people annually and constitute a significant problem to the healthcare system, social stability, and food security, especially in resource-poor countries. Although the major drivers for the emergence and spread of these deadly diseases are urbanisation, climate change and modernisation of the transport industry; poor sanitation and limited access to health services also contribute substantially to the burden of these diseases^2^. Emerging viral diseases are generally categorised into newly emerged viral diseases that have not been known to previously be associated with any disease in man, and re-emerging viral diseases that were recognized previously but have adapted to become major health threats in a population^3^. Unsurprisingly, most emerging viral diseases are caused by RNA viruses which are generally more prone to mutation than their DNA counterparts. The high mutation rate in those viruses, largely driven by genetic recombination events and poor proofreading ability of the viral replicases, is believed to be responsible for the rapid evolution and continued emergence of new often deadly and difficult to control infectious pathogens that quickly adapt to escape vaccine-induced population immunity^4^.

The mucosal route remains the portal of entry for most of these pathogens from where they disseminate further to cause a systemic disease. However, most of the vaccines available against these pathogens are administered systemically. Although this strategy induces a robust systemic immune response, there is often a weak local mucosal immunity which may be inefficacious in curbing mucosal infection. In contrast, the mucosal route of vaccination not only induces a very strong local immunity but also provokes a robust protective systemic response, hence, forming an effective strategy to prevent mucosal colonization^5,6^.

**Fig. 1:**
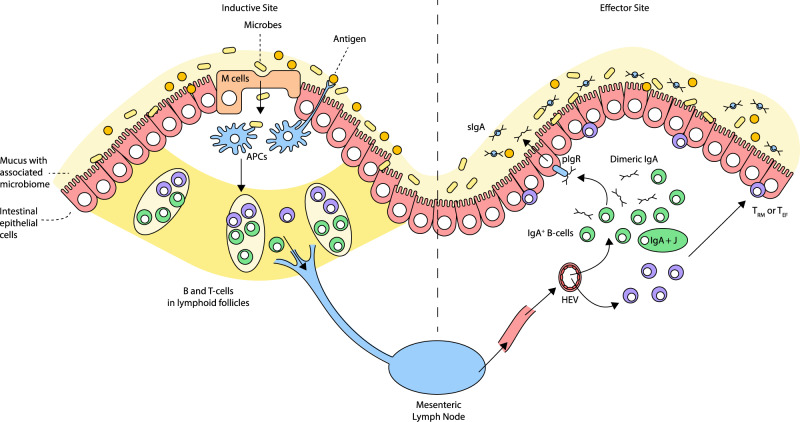
MALT and mucosal immune response. The MALT can be functionally divided into 2 portions, the inductive and the effector sites. The organized lymphoid tissue composed of lymphoid follicles, present along the GIT (GALT) and the respiratory tract (NALT) represent the inductive sites where immune response is initiated. Overlying the follicles are specialized epithelium which in the Peyer’s patches is called the follicle-associated epithelium (FAE). This overlying epithelium is equipped with functionally active microfold cells (M cells) which are involved in antigen sampling from the lumen and delivers these luminal antigens to the underlying DCs and macrophages (APCs) in the subepithelial follicles via transcytosis. Some of the underlying DCs and Macrophages also directly sample antigens from the lumen by the extension of transepithelial dendrites across the epithelium or by occasional migration into the lumen. Following antigen capture, the APCs delivers and present the antigens to the T cells and B cells present in the follicles to induce an antigen-specific immune response. The activated T and B cells then exit the submucosa via the lymphatics to the mesenteric lymph nodes where the immune response may be further exaggerated before finally draining into the systemic circulation. These activated cells then express mucosal homing receptors such as CCR9 and CCR10 and are guarded by gradient of chemokines such as CCL25 and CCL28 present in the mucosa to finally exit the blood, a process mediated by integrins and adhesion molecule α4β7 and MAdCAM-1 respectively. At the effector site where the effector functions are carried out, activated T cells go on to become effector cells and/or tissue-resident memory cells. Activated B cells undergo class-switch to become IgA+ B cells and plasma cells which add joining chains to secrete polymeric IgA. These polymeric IgA are transported transcellular to the lumen following binding to polymeric Ig receptor (pIgR) as secretory IgA (sIgA) which lines the mucus and functions in trapping microbes.

**Fig. 2:**
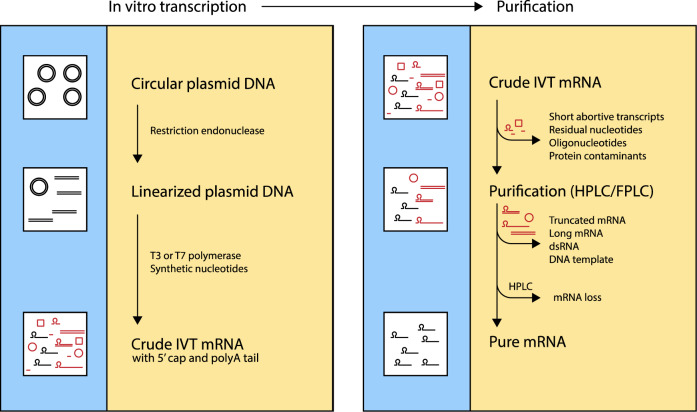
IVT-mRNA transcription and purification. Crude IVT-mRNA which has been generated synthetically from a linearized DNA template using polymerases (T3/T7) often contains a mixture of the mRNA molecules with different categories of impurities. The crude mRNA is initially subjected to extraction and precipitation processes which remove some but not all the impurities, final HPLC or FPLC treatment generates pure grade mRNA molecules.

**Fig. 3:**
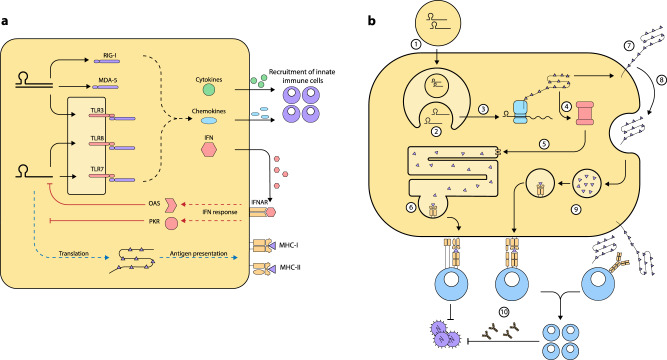
Immune response to mRNA vaccines. **a** Innate Immune response to mRNA vaccines The delivery of the mRNA molecules into the cytosol is followed by the detection of the mRNA molecules by TLRs. Double-stranded RNA molecules co-delivered as impurities or produced from SAM or as secondary structures are additionally detected by RLRs (RIG-1, MDA-5). These drive cytokine and chemokine responses that recruit more innate cells to the injection site. Amplified interferon response can result in the activation of OAS/PKR whose signals impede translation, protein expression and antigen presentation. **b** Adaptive Immune response to mRNA vaccines. Following In vivo delivery of the mRNA vaccine, the mRNA molecules with the delivery vehicles are (1) uptaken by cells such as DCs at the site of injection by endocytosis with subsequent delivery into the endosomes. This is followed by (2) endosomal escape of the mRNA molecules into the cytosol and subsequent (3) translation in the cytosol to produce the encoded protein. The produced protein may be retained in the cytosol where it is subsequently channelled for (4,5,6) proteasomal-MHCI pathway which would eventually drive a CD8 T cell response. Some of the proteins may also become (7) membrane-bound and expressed on the surface or (8) secreted/shed. Some of the secreted or expressed proteins may be (9) recycled by endocytosis and subsequently channelled through the MHCII-restricted presentation to eventually drive CD4 T cell response. B-cell and T-cell responses occur by virtue of their interactions with the secreted or membrane-bound proteins and MHC-antigen complexes respectively. (10) These adaptive responses prevent infection and facilitate the elimination of the pathogen upon encounter through antibody production, cytokine release and cytotoxic activities.

**Fig. 4:**
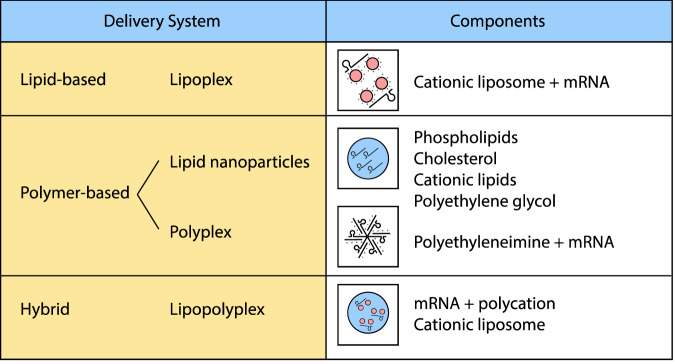
Summary of mRNA delivery systems. This figure provides an overview of the existing in vivo delivery systems and the components of the various classes.

**Fig. 5:**
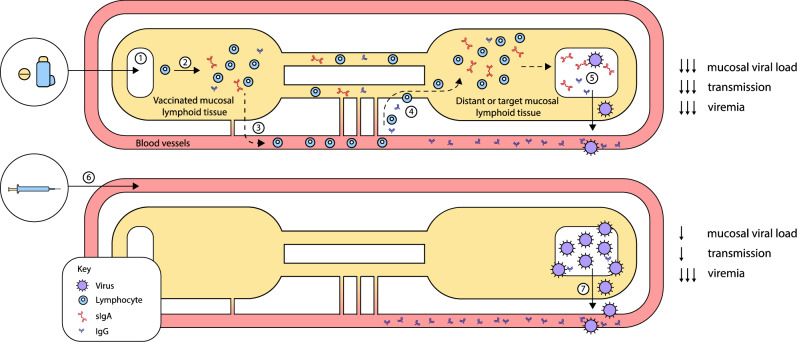
Comparison between mucosal and invasive mRNA vaccination. The immunological benefits of mucosal and invasive vaccine administration as proposed for mRNA vaccines. (1) Following mucosal delivery, the vaccine uptake induces responses at inductive sites in the (2) mucosal lymphoid tissue from where antigen-specific lymphocytes are (3) transported systemically in the blood and home to the primary and distant mucosal surfaces. (4) This results in the production sIgA and the presence of the antigen-specific B and T-lymphocytes at different mucosal sites and eventually preventing (5) mucosal colonization or disease transmission with abundant IgG in the blood to prevent or limit viraemia. In contrast, (6) invasive routes of delivery induce a very effective systemic response with abundant IgG present to prevent viraemia, but (7) this induces a relatively weak mucosal response with little to no sIgA, which may not effectively prevent mucosal infection or transmission.

**Fig. 6:**
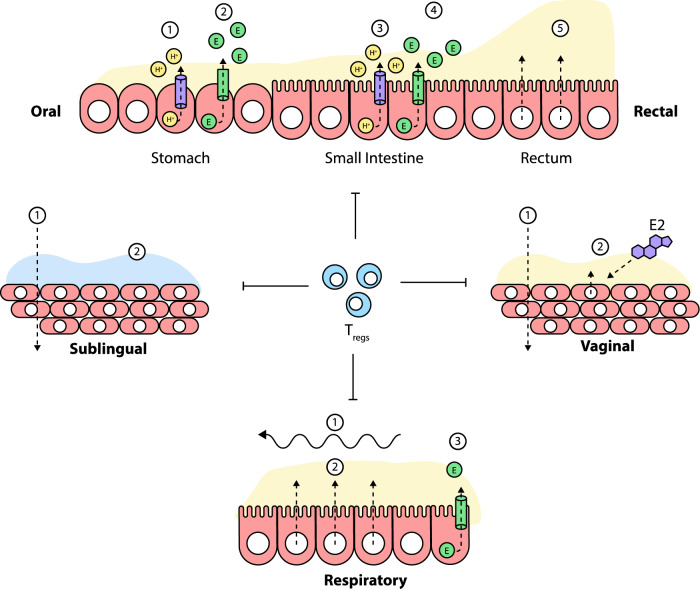
Challenges encountered in the mucosal environment. Following sublingual immunization, the antigen faces challenges associated with the (1) crossing of the stratified epithelium and (2) salivary dilution of antigen. With oral immunization, the vaccine faces challenges in different regions along the GIT. In the stomach, there are challenges due to the (1) destructive action of the acidic environment and (2) the degradative action of enzymes. In the small intestine, the challenges faced are those impacted by the (3) pH variation, (4) degradative enzymes and (5) intestinal mucus in addition to the intrinsic immunotolerance. At the rectal mucosa, the problem faced is those impacted by the (5) thick mucus layer and the tolerogenic microenvironment. Following nasal immunization, at the respiratory mucosa, the vaccine faces challenges due to the (1,2) mucociliary action of the ciliated epithelium which continually pushes the antigen, a process that reduces residence time, the (2) low-grade enzymatic action of RNAses and also the intrinsic immunotolerance. At the vaginal mucosa, the challenges are due to the mucus layer which thickens with increased estradiol (E2) level and reduces uptake as well as the immunotolerance with the low immunogenic response which is often limited to the local genital tract. The epithelial barrier formed by the epithelial layer of cells is a limiting factor to vaccine uptake which is common in all mucosal compartments.

Over the last 3 decades, epidemics of emerging viral diseases including Avian influenza, Ebola, Severe acute respiratory syndrome (SARS), Middle East respiratory syndrome (MERS) and several others have occurred in different parts of the world. The most recent global health threat is Coronavirus disease 19 (COVID-19) caused by the novel corona virus called SARS-COV-2, which emerged in Wuhan city of China in December 2019^7^. Within a few months of its emergence, COVID-19 had spread to all continents on earth, affecting over 265 million people with more than 5.2 million deaths as of 6th December 2021^8^. Indeed COVID-19 has proven to be a global public health emergency that has the potential to ravage the economy and social stability of several countries. So far, the disease has placed millions of people at the risk of falling into extreme poverty, while the number of undernourished people has been projected to be more than 800 million before the beginning of the year 2021^9^. Although most countries took measures such as border closures, trade restrictions and total lock down to curtail the spread of the disease, the actions dramatically disrupted domestic and international food supply chains, aggravating the already existing food insecurity, especially in resource-poor nations. Given this huge socio-economic impact of COVID-19 and indeed all other emerging viral diseases particularly on the most marginalized populations, there is a need to develop effective countermeasures to reduce the burden of emerging viral diseases (Figs. 1–6).

To date, vaccines are considered among the most successful tools for public health interventions against emerging viral pathogens^10^. They have historically been very effective in eradicating or at least reducing mortality and morbidity due to several infectious diseases. Whereas most conventional vaccines have contributed immensely towards infectious disease control, they are developed using strategies that are cumbersome, risky and time consuming, making them unsuitable under pandemic situations where accelerated vaccine development is a priority^11,12^. The recent advances in recombinant DNA technology have paved the way for the emergence of the state-of-the-art “next generation” vaccine technologies. Courtesy of bioinformatics and molecular biology, rationally designed effective vaccines based on synthetic peptides, replication-competent/deficient virus vectors, as well as nucleic acid (DNA and mRNA) have emerged^13^. Among these vaccine platforms, mRNA vaccines have many advantages that make them promising tools for combating emerging viral diseases. First of all, they are simple to generate, safe to handle, easily taken up and processed using the host’s translational machinery^14^. They can also be chemically manufactured at industrial scales without the need for animal or cellular components. Furthermore, they do not need to be trafficked into the nucleus for expression, making them safe from integration into host genome and less susceptible to degradation by enzymes in the nucleus^15^. More so, when intracellularly processed and presented by antigen-presenting cells, the products of mRNA vaccines are able to stimulate robust cell mediated and humoral immune responses in the immunised hosts^16^. mRNA molecules are the templates for protein synthesis in the cytoplasm of a cell. They are however highly unstable, a limitation that has delayed the progress in the application of mRNA technology for infectious disease control. However, with the discovery of strategies for enhancing the stability of mRNA^17^, a growing substantial number of literature now exists that highlights the role of mRNA in modern vaccinology. Finally, these mRNA-based vaccines are being developed for parenteral administrations *via* the intramuscular, intradermal, subcutaneous and intravenous routes^18^. Although these routes have recorded a high degree of success in mRNA vaccinology, the comparative benefits of the mucosal delivery route would be of high value in tackling the viruses at the portal of entry.

## The mucosal-associated lymphoid tissue (MALT)

Numerous studies have outlined the structural and immunological architecture of the Mucosal Associated Lymphoid Tissue (MALT) in man. However, for the purpose of this review, general salient features are outlined while region-specific peculiarities which can influence the uptake of and response to mRNA-based vaccines are discussed. Generally, the mucosal tissue forms the most extensive physical barrier that delineates the internal organs from the physical environment^19–21^. Hence structural and functional adaptations have evolved to maintain a delicate homeostatic balance allowing a large degree of tolerance as well as being reactive towards an invading pathogen. Structurally, the mucosal tissue consists of an epithelial layer covered in mucus which ranges from, simple cuboidal, columnal (simple or pseudostratified), the stratified squamous epithelium (with or without keratinization). These are linked together laterally by tight junctions with junctional proteins, and basally by a basement membrane with associated germ cells or other cells of immunological origin^22,23^. These are then underlaid with lamina propria of loose connective tissue which often houses organized and loose aggregates of lymphoid cells and innate immune cells^22,24^. This general architecture although regionally specialized, affords the mucosal tissue its functional role of ensuring absorption of nutrients, preventing pathogen colonization by providing sentinel functions as well as eliciting defence when the barrier is compromised^24^. The Mucosal Associated Lymphoid tissues (MALT), structures found in the lamina propria, are composed of naive, activated and memory B cells and T cells as well as antigen present cells (Dendritic cells and Macrophages). These are overlaid with specialized epithelia structures called the Follicle-associated epithelium containing functionally active microfold cells (M-cells) which facilitate transduction of antigens (sentinel functions) to the follicles—inductive sites. In fact, the MALT has been described as home to over 80% of effector B-cell population in the body^24,25^. Other inductive sites include tonsils in the palate and adenoids in the oropharynx among others^25,26^. Following antigenic stimulation, class cell-switching and affinity maturation of B cells occur in the germinal centre of the follicles with the associated activated CD4^+^ T cells giving rise to effector cells which migrate to the interfollicular subepithelial area^27^. The interfollicular areas are composed of abundant T-cell population, innate lymphoid cells, along with macrophages, and DCs. An important mechanism of induction-effector distribution of activated cells along different regions of the MALT is influenced by the exiting of activated cells into circulation through the high endothelia venules (HEVs) which then home to mucosal sites guided by adhesion molecules on endothelial surfaces such MAdCAM-1, to elicit effector function^28^. The diffuse areas of the lymphoid population present in the subepithelial lamina propria have been described mostly as the effector sites of the MALT which houses homing activated cells or in-situ activated cellular population (e.g., T-independent activated B cells)^25^. Generally, the MALT consists of bacterial protease-resistant IgA2-producing B-cell population, but this is highest in the large intestine^25,29^. An important adaptation of IgA formed from the MALT is the secretory form (SIgA) which cross links large antigens with multiple epitopes in a neutralization reaction either in the lumen, intraepithelial or at the lamina propria. These SIgAs, formed from complexing of polymeric IgA to its receptor, are trapped in the mucus layer of the MALT which increases in thickness and composition from the oral cavity to the large intestine. The mucus consists of two layers, a thinner inner layer that is sterile and difficult to dislodge and an outer non-sterile layer easy to dislodge^27^. Together both layers consist of mucin 5AC (MUC5AC) and MUC6, produced principally by goblet cells and have a thickness ranging from ~15–400 μm^30^. The MALT is subdivided into different compartments depending on the anatomical location. These are discussed in the following sections.

### The gut-associated lymphoid tissue (GALT)

The Peyer’s patches represent the main inductive sites of the GALT^31^. They are roughly oval structures concentrated in the distal one-third of the ileum although extend into the jejunum with a few numbers in the duodenum identified. This makes the ileum a desirable target for vaccine antigen uptake as both surface area and propensity for uptake is maximized at this site^31,32^. Hence, a vaccine must be designed to withstand the site-specific adaptations of the GI and become easily absorbed at this site. Studies have shown that Peyer’s patches appear as early as the 3rd trimester of life and climaxing in number into the late third decade after which their numbers decrease^31^. The development of Peyer’s patches is also influenced by gut microbiome through diet and environmental influences, hence, making this site a desirable target in infants and young adults. These organized lymphoid tissues of the GALT are composed of abundant number of lymphoid cells, most naive, ranging from B cells, T cells, macrophages and dendritic cells (DCs) including an overlying FAE that contains abundant M cells^32,33^. In addition, double-negative (CD4^**−**^ CD8^**-**^) mucosal-associated Invariant T cells (MAIT) with an invariant TCR which is restricted to an MHC-related 1 (MR1) molecule, have been reported abundant in the GALT. These cells are capable of producing different cytokines in response to microbial invasion and this is largely dependent on the transcriptional factors implicated in the responses^34^. Constituent GALT DCs plays a significant role in the induction-effector function of GI mucosal. Studies have shown GALT-associated MHCII^+^ CD11c^**+**^ CD11b^+^ DCs to be divided into two subsets; the CD103^**+**^ CX3CR1^**−**^ which captures antigen to induce IgA production and imprints immunoregulatory T-cell phenotype, and which is responsible for oral tolerance following the ingestion of protein antigen. The second subset is the CD103^**-**^ CX3CR1^+^ DCs which includes the intestinal macrophages; these cells generally induce an inflammatory response and are responsible for imprinting TH-17 phenotype, being implicated in colitis^35^. Additionally, it was reported that aside the use of transepithelial dendrites extension to capture antigen from the intestinal lumen for indirect antigen sampling, the intestinal CX3CR1^+^ macrophages transiently migrate into the intestinal lumen to capture antigens directly, to trigger cascades of downstream immune response signalling^36^. Sentinel functions in the GI is also carried out extensively by the intestinal epithelia cells armed with vast array of extracellular and intracellular sensors such as Toll-Like Receptors (TLRs)^36^. Indeed, microbiota interaction with receptors on the apical site of IEC mostly triggers tolerogenic pathways orchestrated by TGF-β, BAFF and APRIL from enterocytes and intestinal DCs which drive IgA production in resident B cells^27^. However, stimulation from the microbiota may trigger a potent inflammatory response when there is a breach to the intestinal epithelial barrier due to damages leading to the release of cytokines such as IL-1β and IL-6, and chemokines such as IL-8 (CXCL-8) and RANTES (CCL5). Furthermore, alarm signals can be largely triggered from the apical IECs thereby breaking the tolerance threshold following the interaction of microbial structures (such a Lipopolysaccharides component of the bacterial cell wall) with PRRs on the extracellular or intracellular milieu of the GI epithelium^37^.

### Nasopharynx associated lymphoid tissue (NALT)

The NALT has been described as the main inductive site of the respiratory mucosa, being the equivalent of Peyer’s patches in the gut in its structural organization. Generally, the NALT includes the organized lymphoid structures in the nose, the bronchial and the larynx. This also encompasses the oropharyngeal lymphoid structures such as the palatine tonsils and the adenoids which altogether form the Waldeyer’s ring. The organized lymphoid structures houses immune cells for inducing T and B cell responses^38^. All the aforementioned lymphoid structures of the respiratory system develop first in early life and persist beyond childhood^39^. This phenomenon can possibly be attributed to the induction of antigens in breathed air passing through the nares before getting to the lungs. Therefore, in a similar vein, vaccination strategies targeting the NALT in the nasal epithelium at the precise particle size could stimulate uptake induction and elicit protection against a respiratory pathogen that opportunistically wants to colonize the nasal mucosa. This would be feasible by understanding and exploring the antigenic inductive mechanisms of the NALT in its unique form. Several lymphoid and innate cells, e.g., T cells, B cells, macrophages, and DCs, have been reported to be present in the NALT and are involved in the response not only to invading pathogens but also to vaccine antigens^40,41^. Similar to the GALT, the DCs in the NALT are separated from the nasal cavity by a FAE lined with M cells which facilitate the transfer of antigens to the underlying lymphoid structures^33,42^. The airway is lined by specialized epithelium which functions in response against microbial invasion. The epithelial cells are equipped with PRRs which can sense microbial ligands to trigger the release of cytokine and chemokines that stimulate immune cells in the NALT. Additionally, airway epithelia cells have been reported to produce BAFF (B Cell-activating factor of the TNF family) and APRIL (A Proliferation Inducing Ligand) following TLR-3 activation which alongside CD40 activates B cells in a T-dependent interaction to induce class-switching to IgA+ B cells that produce sIgA^43^. It has been reported that CD8 + resident memory T cells (T_RM_) are generated by local antigen production at the respiratory mucosa following MHC-I restricted antigen presentation by stromal cells and migratory CD103 + DCs. These non-migratory cells remain in the lung tissue and serve to respond during secondary infection, acting in both innate and adaptive fashion, owing to prestored cytokines such as IFN-γ prestored at the transcriptomic level^44^. Indeed, targeting the development of these cells by intranasal immunization could be beneficial in controlling respiratory viruses.

### The oral cavity and the sublingual mucosa

Although the oral cavity is near the nasal cavity, it is lined with squamous stratified epithelium which is a functional adaptation to withstand insults, prevent penetration of xenobiotic agents and in turn contains diffuse lymphoid tissue as inductive machinery. Some areas of the buccal mucosa are keratinized such as the gingival, hard palate and the outer lips thus adding extra layer of imperviousness against insults and of course, antigenic uptake^45,46^. For this review, sites of possible antigenic induction, specifically the sub-lingual area will be discussed. This is due to the ease of accessibility and lack of keratinized epithelium which makes antigenic uptake easier than other sites of the oral cavity such as the gingivae, tonsillar areas and cheeks. However, this region is continuously bathed with saliva, a feature that has limited drug and vaccine delivery through this route^46^. When compared to the respiratory mucosa, the sublingual mucosa has about 5 times relatively lesser surface area. In addition, the sublingual mucosa lacks antigen-sampling M cells, Follicular DCs and is devoid of organized lymphoid follicles. Nonetheless, several cellular populations have been reported in the sublingual epithelium ranging from DCs of different phenotype, to eosinophils, macrophages, mast cells, tissue-resident memory T cells, B lymphocyte and natural killer cells. In addition, CD11b^+^, CD11c^+^ EpCAM^+^ CD201^+/−^ oral Langerhans cells possessing TLR 2,3,4,9 have been reported to be present—a feature that increase antigen uptake and makes this route desirable for vaccine administration^47,48^.

### The vagina mucosa

The reproductive tract has received a relatively lower attention in mucosal immunity compared to the other mucosal routes. This region differs with respect to the innate and adaptive immunity, mucus production and microbiota composition. Also, the vaginal mucosal epithelium is stratified compared to the monolayer found in the intestinal or lung mucosa^49^. The upper reproductive mucosa (type-I mucosa) is enriched in sIgA while the lower reproductive mucosa (type-II) which encompasses the vagina and ectocervix lacks sIgA and are devoid of MALT. The region between the type-I &II mucosa is the transformative zone, which is rich in T cells and APCs, this zone also represents a frequent site of vaginal infection. The vaginal mucosa is covered by thick mucus which helps in trapping pathogens. The thickness of mucus and the composition of both immune and non-immune cells is influenced by hormonal changes during the menstrual cycle. Generally, the submucosal region of the type-I epithelium is rich in DCs, macrophages and memory lymphocytes while that of the type-II epithelium consists of a sparse network of DCs, macrophages and a few lymphocytes. The uterine epithelium has a lymphoid aggregate of B cells and CD8^+^ T cells which becomes enlarged during infection^50^. Following the induction of vaginal infection, the effector site is limited to the local reproductive mucosal immunity. This local vaginal immunity has been shown to be particularly important in providing full protection against HPV, HIV and HSV. Both neutralizing antibody (HPV) and T cells (HIV and HSV) are relevant in this context. This involves both CD4+ and CD8+ T cells as the former are required to drive a robust CD8 T cell response that mediates cytotoxicity of HIV-infected cells. Thus, the vaginal mucosa should be explored for vaccine design against sexually transmitted diseases^49^.

## The mRNA vaccine platform

### Considerations in design and construction

Synthetic mRNA vaccines are designed based on the blueprint of eukaryotic mRNA^51^. There are basically three types of mRNA vaccines; linear conventional non-replicating mRNA, circular RNA and virally derived self-amplifying linear mRNA vaccines^52,53^. Of these, the two linear mRNA types are popularly employed in infectious disease vaccine applications. The two types have four essential features in common: a cap structure, 5′ and 3′ untranslated regions (UTRs), an open-reading frame (ORF), and a 3′ poly (A) tail. An additional structure which is unique to the self-amplifying mRNA constructs is the genetic replication machinery usually derived from Alphaviruses^54^. This replicase system is together formed by the four non-structural proteins (nsp), viz, nsp-1 to nsp-4, all of which are encoded in the alphavirus genome^55^. The 5′ cap and 3′ poly (A) tails are crucial for enhancing the translational efficiency and transcript stability of the mRNA vaccine. In particular, the 5′ 7-methylguanosine (m7G) cap of the mRNA molecule plays a significant role in translation by blocking the 5′–3′ exonuclease-mediated degradation^56^. Similarly, poly (A) tail at its optimal length, may enhance translation and provide protection to the mRNA.^57^. The 5′ and 3′ UTRs are critical regulators of protein translation; hence, the choice and construction of these UTRs are phenomenal for the optimal antigen expression and vaccine efficacy^58^. The coding sequence of the gene of interest may also be codon-optimised to synonymously substitute the rare codons with those codons whose tRNAs are abundant in the cytoplasm in order to achieve optimal production^59^. The mRNA construct can be modified by including codons such as pseudouridine, 2-thiouridine and 5-methylcytidine into the mRNA in a nucleoside-modified mRNA vaccine. This approach has been shown to reduce innate recognition by PRRs and decrease the degradation of the mRNA molecules thereby increasing stability and translation efficiency^59–61^. Given the role of the mRNA structural elements in determining vaccine efficacy, careful considerations must be made in designing and constructing engineered mRNA vaccines.

### Purification for optimal expression

Following the synthesis of the mRNA from the plasmid DNA, the resultant mRNA is often in complex with impurities which must be removed to liberate a purified therapeutic mRNA which is suitable as a vaccine candidate. The first purification step is to get rid of other reaction components which are in complex with the mRNA transcript by a series of extraction and precipitation processes. The sample is left with shorter than normal mRNA arising from premature termination of the elongation step as well as longer than normal mRNA arising from linearized or non-linearized template DNA^14^. Furthermore, the sample contains other contaminants such as residual DNA templates, enzymes and double-stranded RNA transcripts^55^. An effective and efficient purification of the mRNA to remove all these contaminants is crucial to generate a high-quality mRNA before it can be considerably utilized as a therapeutic substance. The elimination of the double-stranded RNA transcripts is of particular importance as these can activate intracellular RNA sensors which triggers IFN-I production and reduces the downstream antigen-driven adaptive response and efficacy of the mRNA vaccine^62^. In addition, purification of the mRNA is essential for optimal and efficient expression of the encoded protein which would enhance a sustained antigen availability, presentation, and downstream adaptive response. Following purification, the mRNA is further subjected to sterility, potency, identity, and purity testing before being finally formulated into a therapeutic product of Good Manufacturing Practice (GMP) grade. Thermal stability is a considerable issue with mRNA vaccine formulation as there is need to preserve an extensive cold chain from the development, transportation, distribution, and storage at all points along the chain. This remains a great disadvantage particularly for the developing countries where power supply is limited, and the cold chain maintenance is uncertain for distribution and storage of the vaccines^63^.

### Immunogenicity and efficacy

The immunogenicity of mRNA vaccines is driven mainly *via* the expression of the encoded protein by utilizing the host cellular translational machinery thereby triggering robust cellular and humoral adaptive responses. The encoded protein antigen can be secreted, membrane-bound or retained in the cytosol depending on the mRNA sequence delivered into the intracellular milieu. This sequence may be intrinsic to the primary antigenic protein or engineered to either or not include both signal peptide and transmembrane domain sequences^55^. Although the innate immune cells are the desired target of mRNA uptake following in vivo delivery, however, there is possibility of uptake of the mRNA formulation by the cells at the site of injection which are non-APCs and since these cells possess MHC-I, they may translate and express the antigen in MHCI context. This off-target expression may result in cytotoxic T cell killing and ADCC, hence, efforts should be made to direct mRNA molecule to DCs to avoid off-target toxicity, improve uptake and enhance efficacy^64^. Similar to viral genomes, mRNA can be detected by intracellular nucleic acid sensors such as TLR7 and TLR8 found in antigen-surveillant DCs, monocytes and macrophages^59^. These innate responses have been reported to contribute to the success of mRNA vaccines by driving both cytokine and chemokine responses which induce adaptive cellular and humoral response against the encoded protein antigen^55,65^. Furthermore, the immunogenicity of mRNA vaccines can be linked to the provocation of both MHC-I and MHC-II restricted antigen presentation.

Being recognized by intracellular RNA sensors, exogenous mRNA has intrinsic immunostimulatory activity making it self-adjuvanting, this property can however have both beneficial and detrimental effect on the potency of the mRNA vaccine by triggering type-I IFN response with downstream immune response on the one hand and impairing translation through the activation of 2′−5′-oligoadenylate synthetase (OAS) and protein kinase R (PKR) respectively^60,66–68^. Apart from optimal purification, there are approaches which have so far been shown to further dampen the immunostimulatory activity and reduce the innate sensing of the mRNA thereby allowing a more efficient translation and increasing the immunogenicity of the mRNA construct, these are extensively covered in other reviews^52,55,60,66,68–70^. Self-amplifying mRNA (SAM) constructs are another strategy for increasing encoded antigen expression and improving immunogenicity at a comparatively lower dose than non-replicating mRNA since this can multiply in the intracellular milieu, a detailed discussion of the RNA species is covered in other reviews^55,71–73^.

### In vivo delivery systems for optimal translation and expression

An efficient delivery system is crucial for the success of mRNA vaccines to reach their full potential in terms of translation, immunogenicity and efficacy. It has been shown that expression occurs and peaks within the first 4 hours following intravenous administration of mRNA vaccine and this may persist up to 10 days after intramuscular or intradermal administration^74^. Many strategies have been explored for in vivo delivery of mRNA into target cells. These strategies aim at ensuring the protection of the mRNA molecules from degradative actions of nucleases, maximal delivery of the mRNA into cells, effective translocation into the cytosol from the endosomal compartments and improving the overall translation, protein expression as well as immunogenicity of the mRNA. These strategies include the use of lipid-based, polymer-based or hybrid carriers as delivery vehicles. The most commonly explored delivery vehicle with a track record of high efficiency is the use of lipid-based carriers which may be in form of lipoplexes or lipid nanoparticles (LNP)^52^. Lipoplexes are complexes formed by the electrostatic interactions between cationic liposome carriers and RNA molecules. Liposomes are nanosized spherical vesicular structures made of amphiphilic phospholipid bilayers composed of a hydrophilic head and two hydrophobic tails with an aqueous core. The spherical structures assemble when the phospholipid bilayer is present in an aqueous environment, whereby the hydrophilic head tend to interact with the aqueous environment while the hydrophobic tail tend to interact with one another^75^. They have been used as vehicles for gene delivery, silencing-RNA delivery and have been explored in both cancers and infectious diseases for mRNA vaccine delivery. This is due to the high transfection rates of liposomes compared to other non-viral vectors, their biodegradability and ease of synthesis. In the field of IVT mRNA delivery, cationic lipids like 1,2-di-O-octadecenyl-3-trimethylammonium-propane (DOTMA) and 1,2-dioleoyl-3-trimethylammonium propane (DOTAP) have been explored as well as 1,2- dioleoyl-*sn*-glycero-3-phosphoethanolamine (DOPE) which is a zwitterionic lipid^69,76,77^. Ionisable lipids which are neutral at physiological pH but become cationic in an acidic pH remain viable alternatives to cationic lipids offering a relatively reduced toxicity and facilitate endosomal escape of antigens for cytosolic delivery^78^. LNP-based delivery systems are the most used carrier vehicles at present. Many studies have reported their successes in both infectious diseases, e.g., zika virus, influenza virus, HIV and rabies virus infections as well as in cancers. Notably, LNPs have so far offered the best results. The basic components are cationic/ionisable lipids, cholesterol, polyethylene glycol (PEG) lipids and phospholipids; these work synchronously to achieve efficient protection, uptake, endosomal escape, and translation of mRNA molecules to drive robust cellular and humoral responses^52,55,78–80^. Polymer-based and hybrid delivery systems are extensively discussed in other reviews^52,68,77^.

## mRNA vaccines and emerging diseases

### The mRNA vaccines against covid-19

In the quest to end the current COVID-19 global pandemic, there have been concerted efforts worldwide to develop safe and effective vaccines giving rise to many vaccine platforms being developed and trialled in different parts of the world. This is particularly important owing to the fact that there is no specific therapeutic approach against the disease which led experts to reach consensus that safe and effective vaccines are crucial in order to put an end to the pandemic^81^. These efforts have led to an accelerated development of many next-generation vaccines such as viral vectored vaccines, DNA vaccines, antigen-presenting cells and mRNA vaccines. So far, two mRNA vaccines have been approved for use clinically in many parts of the world, although there have been other next-generation vaccines currently in-use worldwide^82^.

There are seven mRNA COVID-19 vaccine candidates in clinical trials as of January 2021 representing only 11% of the total 63 COVID-19 vaccine platforms in clinical trials. Out of these, only 2 mRNA-based vaccines being approved for human use so far^83^. The mRNA vaccines currently in use were developed by Moderna (mRNA-1273) and Pfizer-BioNtech (BNT162b) US companies. Both vaccines consist of a nucleoside-modified mRNA encoding the SARS-CoV2 spike protein with two proline mutations in the sequence at positions 986 and 987 to keep the encoded protein locked in the pre-fusion conformation. This modification is necessary to mimic the native three-dimensional conformation of the SARS-CoV2 spike protein just before binding to the ACE2 receptor of host cells. The mRNA is nucleoside-modified by uridine replacement with 1-methylpseusouridine to reduce innate responses as discussed earlier. The delivery vehicle used in both cases is the LNP-based vehicle and both vaccines are administered parenterally in 2 separate doses 28 days apart and 21 days apart for Moderna and Pfizer vaccines respectively. The immunogenicity and safety profiles have been good for both vaccines; however, the Pfizer vaccine reportedly has a slightly higher efficacy than the Moderna vaccine with a record efficacy profiles of 94.5% and 95% for Moderna and Pfizer vaccines respectively^81,82,84^. Overall, the two vaccines have shown protective cellular and humoral responses targeted towards the SARS-CoV2 spike protein with the T-cell response biased towards the Th1 response. Antibody induction is reportedly broadly neutralizing with titres higher than that of human convalescent serum from COVID patients. There is no significant difference in the humoral response of the elderly when compared to that of the younger population; this is of particular importance since the older populations develop more severe diseases^82,85^. However, recent vaccine effectiveness studies revealed a reduced effectiveness in the elderly (≥65 years) compared to younger adults (18–64 years) with an adjusted VE of 79.8% and 95.1% respectively^86^. The main disadvantage of these two vaccines is that they have to be stored at a very low temperature to ensure the stability of RNA along the supply chain. Despite its higher efficacy and lower cost, the Pfizer vaccine initially required a much lower storage temperature of around −80 to −60 °C and poses more storage challenges as compared to the Moderna vaccine which can be stored at a relatively higher temperature of about −25 to −15 °C which is more feasible. More recently, a warmer temperature storage between −25 and −15 °C has also become feasible for Pfizer vaccine in a duration of up to 2 weeks following which the vaccines can be returned to the ultracold temperature for a more prolonged shelf life^87^. Both vaccines have been reported to cause mild adverse reactions such as soreness, redness or pain at injection sites, fever, headache and rare anaphylaxis. Overall, the incidence of adverse events is lower in the Pfizer than the Moderna vaccine^84^. Vaccine effectiveness (VE) studies to assess the real-world impact of these vaccines have also revealed positive impact in preventing hospitalization and severe diseases. A study involving 21 US hospitals revealed an effectiveness in preventing hospitalization standing at 88% and 93% for Pfizer and Morderna vaccines respectively after a 2-dose schedule. This was relatively higher than that obtained for the Jansen vaccine which was 71% effective in preventing hospitalization. Apart from the fact that the Morderna vaccine has a higher VE than the Pfizer vaccine, this study also noted a decline in the VE of Pfizer vaccine within 120 days. A higher anti-RBD antibody was also reported for Morderna than the Pfizer vaccine. These differences in VE were possibly linked to the amount of mRNA particles in the Morderna vaccine and a longer interval of 28 days between doses as compared to the 21 days of Pfizer^88^. Another study reported a lower overall VE of 86% for immunocompromised individuals in reducing hospitalization and severe diseases as compared to immunocompetent individuals after 2-dose vaccination with either Morderna or Pfizer^89^. However, these vaccines are less effective in preventing infection especially in the face of evolving viral strains.

In addition to the approved mRNA COVID-19 vaccines described above, the other mRNA-based COVID-19 vaccines under clinical studies are summarized in Table 1.

**Table 1:** mRNA-based COVID-19 vaccines in clinical trials.

Vaccines	Developers	mRNA type	Delivery system	Encoded protein	Route	Trial phase	References/Trial number
CVnCoV	Curevac/Bayer	Conventional	LNP	FL pre-fusion Spike	IM	III	^91^
							ClinicalTrials.gov/ NCT04674189
ARCT-021	Arcthurus Therapeutics	SA	Lipid-based	VEEV-FL-S	IM	II	^83^
							ClinicalTrials.gov/ NCT04668339
LNP-nCoVsaRNA	Imperial College London	SA	LNP	VEEV-FL-S-2P	IM	I	^83,161^
ChulaCov19 mRNA vaccine	Chulalongkorn University	Conventional	LNP	NA	IM	I/II	^161^
							ClinicalTrials.gov/ NCT04566276
ARCoV	AMS/Walvax	Conventional	LNP	RBD-S	IM	I	^51^

Other mRNA COVID-19 vaccine strategies with the delivery systems at different phases of clinical trials: *FL* full-length, *IM* intramuscular, *LNP* lipid nanoparticles, *NA* not available, *RBD* receptor binding domain subunit, *S* spike protein, *SA* self-amplifying, *VEEV* Venezuelan equine encephalitis virus.

### mRNA vaccines in other viral diseases

mRNA vaccines are potentially beneficial as compared to other conventional vaccines in the fight against emerging infectious diseases as they can be rapidly developed and optimized in a scalable manner. This enables them to be rapidly directed against the most rapidly emerging and re-emerging pathogens, most especially viruses, which are potential causes of epidemic and pandemics^52^. In contrast, conventional vaccine development is rather time-consuming which could take several years to develop and often complicated by scale-up and commercialization issues while mRNA vaccines can be effectively developed within a shorter time frame to meet large-scale demand. Furthermore, mRNA vaccines can be rapidly modified and developed to be efficiently directed against emerging and fast-mutating pathogens in response to outbreaks since they are not prone to issues of pre-existing immunity which remains a huge challenge to the success of viral vector-based vaccine^90^. There are several pre-clinical studies of mRNA vaccines focusing on emerging viral diseases such as Zika virus, HIV, CMV, Influenza virus, Ebola virus and Rabies virus with encouraging results in terms of safety and effective immunity conferred by both CD4 and CD8 T cells, as well as neutralizing antibodies. Some have subsequently been translated to clinical trials in human subjects^68^. Although both non-replicating and self-amplifying vaccines against viral diseases have been subjected to pre-clinical and clinical studies, the conventional mRNA vaccines have so far progressed furthest in clinical practice^91^. Summarized in Table 2 are some of the most important virus-directed mRNA vaccine trials in either preclinical or clinical studies.

## Exploring the different MALT compartments—mucosal routes of vaccination

The concept of mucosal vaccination was borne out of the realization that greater than 90% of the pathogen that infect human enters the human system via the mucosal routes and the limitation of the current injection-based immunization in substantially controlling mucosal infection and systemic dissemination of these pathogens^5,92,93^. In general, the mucosa has a highly organized immune system thereby rendering it an important target for vaccination^93,94^. This is reinforced by the fact that priming of DCs at a specific mucosa largely determine the homing of T cells to the same mucosa and other distant mucosal sites to fight pathogens at these entry portals^95,96^, based on the principle of common mucosal immunity. There are different routes of mucosal vaccination which induce varying degree of vaccine-specific immune responses that in-turn determines the vaccine efficacy. Mucosal vaccines have been explored through the oral, nasal, sublingual, ocular, rectal and vaginal routes of administration. However, the oral and nasal routes have been mostly employed since these induce a broader mucosal and systemic responses compared to the other mucosal routes^93,96^. The nature of the antigen, the targeted mucosal tissue and the desired site of the induced immune response largely influence the selected routes of vaccination^97^. Since different mucosal routes induce different level of responses in terms of potency and longevity, this reflects the variation in the cellular make up and the organization of the mucosal lymphoid tissues at the different mucosal sites. Hence, it is expected that the same vaccine/antigen administered through different mucosal routes would result in varying degree of protective immunity^93,98^. For example, it has been shown that vaccination against *Mycoplasma gallisepticum* induces a better effectiveness via the ocular route than the nasal route, whereas, the oral route of administration has a relatively negligible vaccination outcome^99^. This is so because effector T cells and B cells preferentially home to the inductive mucosa compartment and the neighbouring anatomical sites. The route of vaccination needs to be carefully considered depending on the mucosal tissues targeted by the pathogen in question.

### The nasal route

The intranasal route has been shown to induce robust cellular and humoral response at the respiratory tract against different pathogens following vaccine-specific induction of the nasal immune system/NALT^100^. In addition, nasal vaccination effectively induces response in the bronchial-associated lymphoid tissue (BALT), the urogenital tract, the salivary gland and the gastrointestinal tract^93,101^. Intranasal administration of influenza vaccine (Flumist) has been shown to display a superior protective immunity than the intramuscularly administered counterpart with effective IgA production at the nasal epithelia^94,102^. In addition, this has been reported to provide cross-protection against seasonal influenza strains including genetically drifted strains^103^. Furthermore, the intranasal route has been explored to induce protective immunity at the GIT against shigellosis. Preclinical and clinical trials revealed a potent GI protection following intranasal vaccination against *Shigella flexneri* thereby further indicating the crosstalk between the different mucosal sites^104^. In addition, the crosstalk between the nasal and genital mucosa is being researched to tackle genital infections and sexually transmitted diseases via intranasal vaccination. Since the nasal route of vaccination induces robust antibody response in the vaginal mucosa, this strategy has been explored with success in HIV research. Here, preclinical studies in rhesus macaques showed the induction of protective IgA and IgG at the vaginal mucosa against HIV-1 infection and subsequent prevention of HIV transmission via the genital tract after an intranasal immunization with a trimeric HIV-1 gp41 protein grafted on a virosome^105^. However, this strategy induced a relatively low T-cell response at the vaginal mucosa as compared to the direct vaginal vaccination, hence, a synergistically broader response encompassing both humoral and cellular anti-HIV response was achieved by combining intranasal immunization with a local vaginal vaccination in a prime-boost technique^106^. This technique is further necessitated by the failure of the vaginal immunization with HIV gp140 to induce protective immunity in healthy women in a clinical trial^107^. Therefore, apart from the potential use to control respiratory pathogens, mRNA vaccines can be targeted in a prime-boost immunization strategy through intranasal and intravaginal administration to tackle HIV, HPV and other STDs. In a recent study, a parenteral prime immunization with mRNA SARS-CoV-2 vaccine followed by an intranasal boosting with an adenovirus-vectored (mRNA prime-Ad boost) vaccine significantly induced a very good IgG, IgA and T_RM_ at the respiratory mucosa. When compared with a parenteral DNA prime-intranasal Adenovirus boost, the mRNA prime-Ad boost strategy had a more efficient neutralization of the virus variant of concern and a more comprehensive T cell response comprising of both circulating T cells and T_RM_^44^. Indeed, the nasal route of vaccination offers several advantages. Apart from the induction of broad humoral and cellular responses, the nasal immunization strategy avoids first-pass metabolism due to abundant venous blood that delivers antigens directly into the systemic circulation, limits the risk of anaphylaxis, offers a very large surface area, and avoids the harsh PH and the excess degradative enzymes attainable at the oral mucosa. In addition, this route offers a dose-sparing advantage. However, this route poses the risk of rapid antigen clearance due to the mucociliary actions leading to short nasal residence time. There is also a poor uptake of soluble antigens and antigen uptake may be impaired in patients with respiratory problems^94,101^.

**Table 2:** mRNA vaccines against some emerging/re-emerging viruses.

Emerging viruses	Encoded antigen	Delivery system	Route	Protective response	References
HCMV	gB, PC, pp65	LNP	Intramuscular	T cells and Neutralizing antibodies	^162^
Rabies	Rabies virus glycoprotein	Protamine	Intramuscular and intradermal	T cells and Neutralizing antibodies	^163^
Ebola	Envelope glycoprotein	LNP	Intramuscular	Neutralizing antibodies	^164^
Zika	prM-E	LNP	Intramuscular	Neutralizing antibodies	^165^ ClinicalTrials.gov/
					NCT03014089
Influenza	Hemagluttinin glycoprotein (H10N8 and H7N9)	LNP	Intramuscular and intradermal	T cells and neutralizing antibodies	^166^
HIV	HIV-1 Tat, Nef and Rev proteins	Autologous dendritic cells	Subcutaneous and intradermal	T cells	^167^
RSV	Fusion protein	LNP	Intramuscular	T cells and neutralizing antibodies	^168^
Dengue	PrM-E	LNP	–	T cells and neutralizing antibodies	^169^
Chikungunya	CHIKV structural polyprotein	–	Intramuscular	Neutralizing antibodies	^170,171^ ClinicalTrials.gov/
					NCT03325075

Other mRNA vaccines against emerging/re-emerging viral diseases in preclinical and clinical development: *CHIKV* chikungunya virus, *gB* glycoprotein B, *HCMV* human cytomegalovirus, *HIV* human immunodeficiency virus, *PC* pentameric complex, *LNP* lipid nanoparticles, *PC* pentameric complex, *prM-E* pre-membrane & envelope.

**Table 3:** Currently licensed mucosal vaccines.

Pathogen	Nature of vaccine	Trade name	Route of administration	Dosage form	Protective immunity	Vaccine efficacy
Poliovirus	Live attenuated polioviruses serotype 1 & 3	Biopolio oral polio vaccine (bPOV)	Oral	Liquid	Systemic IgG and mucosal IgA	Above 90% in most part of the globe
*Vibrio cholerae*	Inactivated *Vibrio cholerae* with CTB	Duchoral	Oral	Liquid	CTB-specific antibody, gut antitoxin, mucosal IgA and anti-LPS antibodies	Strong gut protection above 85%
*Vibrio cholerae*	Inactivated *Vibrio cholerae*	Shanchol, Euvicol	Oral	Liquid	Gut antitoxin and mucosal IgA, anti-LPS antibodies	Strong gut protection above 85%
*Vibrio cholerae*	Live attenuated	Vaxchora	Oral	Liquid	Vibriocidal antibodies^172^	Variable but generally above 70%^172^
*Salmonella typhimurium*	Live attenuated	Vivotif	Oral	Enteric coated capsule	Mucosal IgA, systemic IgG and CTL	Above 50% but variable
Rotavirus	Live attenuated	Rotarix	Oral	Liquid	Mucosal IgA and systemic IgG neutralizing antibody	70–90% (severe disease)
Rotavirus	Live reassortant	Rotateq	Oral	Liquid	Mucosal IgA and systemic IgG neutralizing antibody	70–90% (severe disease)
Influenza virus	Live attenuated	Flumist	Nasal	Spray	HA and NA-specific mucosal IgA and systemic neutralizing IgG. CTL probable	Variable in adult, above 85% in children
Adenovirus^a^	Live attenuated type 4 & 7	–	Oral	Enteric coated tablet	Serum neutralizing antibodies^173^	–

A summary of the mucosal vaccines platform with the specific routes and the induced immune responses. Adapted from refs. ^2,33,92,93,95,122^. *CTB* cholera toxin subunit B, *CTL* cytotoxic T-lymphocytes, *HA* hemagglutinin, *IgA* immunoglobulin A, *IgG* immunoglobulin G, *LPS* lipopolysaccharide.

^a^Licensed for use in the military.

### The oral route

The oral route of vaccination is capable of inducing robust immune response at the GIT, salivary gland, mammary gland as well as the NALT^93^. Along the GI system, effector immune cells are preferentially home to the lamina propria, salivary gland, stomach and intestinal epithelium with a robust sIgA response. In addition to the broad mucosal immunity, the oral route has been shown to elicit a systemic neutralizing IgG response^92,94^. The oral route remains the most patient-friendly route of therapeutic intervention with over 60% of pharmaceutical drugs currently being administered orally. However, the success of an oral vaccination depends on the effective delivery of the antigen to the intestine, crossing of the intestinal epithelium and subsequent activation of the intestinal APC^5^. This makes the oral route the most challenging in terms of vaccine development partly due to the harsh mucosal environment which rapidly degrades the antigen and partly due to the immunological tolerance which fails to induce protective immunity, in addition to the mucus barrier and flexible residence time determined by gastric emptying. This has led to several experimental attempts of oral vaccination which failed to make it past clinical trials due to disappointing results^5,92^. Nevertheless, there exist some oral vaccines with good clinical efficacy; these include the vaccines against polio, rotavirus and cholera. These vaccines are licensed by the WHO and remain a good example for the outstanding efficacy of oral vaccination giving a strong prospect for oral mRNA vaccination^94^. The oral route also offers many advantages over other routes. It is a relatively safe and cost-effective route allowing for self-administration thereby increasing vaccine acceptance and facilitating vaccine distribution. Furthermore, there is no risk of needle-stick injury or local injection-site reaction^92,94,95^.

### The rectal route

Although the oral and nasal routes are the most explored, researchers are now exploring other mucosal routes such as the sublingual, rectal and the intravaginal routes. The rectal immunization route induces protective response at the rectal mucosa, the nasal secretion and the tears. This route is suited for pathogens which enter through the GIT and particularly those adapted to the lower GIT^93,94^. The administration of a peptide-vaccinia prime-boost vaccine via the intrarectal route in macaques was shown to elicit a high avidity CD8 T-cell response at GI mucosa and this correlated with a lower dissemination of SIV^108^. In addition, earlier studies have shown that intrarectal immunization was more effective than the subcutaneous route in the control of SIV viraemia or mucosal infection and the induction of CD8 T cells in macaques^109^. Perhaps, this route may be explored for mRNA vaccine delivery against HIV.

### The sublingual route

The sublingual has gained a lot of research attention in recent years. This route has been shown to also induce a very broadly disseminated mucosal and systemic responses. Studies have also depicted a robust systemic IgG and mucosal IgA induction as well as CD8 T-cell response following sublingual vaccination^110,111^. Furthermore, a comparison of the sublingual vaccination with intranasal, transdermal, intravaginal and intramuscular vaccination using the HPV 16 L1 protein depicted a more robust immunity from the sublingual routes than the other routes^111^. This route has comparable effectiveness to the nasal route in terms of the breadth of the induced response and in contrast to the nasal route, there is no risk of perturbation of the CNS by the redirection of antigens or adjuvants^112^. Since the antigen is directly administered via the oral mucosa into the bloodstream, the sublingual route bypasses the action of proteases and the harsh GI environment, thereby, making it a very promising route of mucosal administration^94^. The sublingual route has equally been shown to induce protective immunity in the lungs and the GIT. A study depicted a protective response against influenza virus challenge in mice following sublingual vaccination with a live attenuated or formalin-inactivated influenza virus^113^. Sublingual vaccination with a recombinant Helicobacter lipoprotein provides a protective immunity against *Helicobacter pylori* in the stomach, more so, this route was better than intragastric vaccination in terms of the protective response^114^. Hence, provided a perfect delivery vehicle can be constructed, this route has a potentially wide applicability for mRNA vaccination against a wide variety of pathogens.

### The vaginal route

The vaginal route is a potentially viable route for sexually transmitted diseases such as HIV. The vaginal mucosa has a large surface area which has a relatively low level of degradative enzymatic activity with a possibility for self-administration of vaccines^94^. However, this route may induce a relatively weak mucosal response which is most probably limited to the local vaginal mucosa. Furthermore, immunization with live or vector vaccines induce effective mucosal response while vaccination with inactivated or subunit vaccines have shown poor responses in mice^96^. For example, it was depicted in a study that intravaginal immunization with *Salmonella enterica* expressing the HPV16 L1 protein induced a protective mucosal and systemic humoral and cellular responses which were protective against HPV and prevent the development of subcutaneously transplanted HPV16 tumour^115^. Furthermore, the intravaginal route can be explored as an adjunct to other immunization routes to improve vaccine response. For example, intravaginal administration of TLR3 and/or TLR9 agonists following subcutaneous immunization with HPV E7 antigen lead to about fivefold increase in the level of INF-gamma-producing CD8 T cells homing to the vaginal mucosa with a threefold higher level of tumour regression of HPV16 tumour compared to only subcutaneous immunization in mice^116^. However, it is worth noting that intravaginal immunization may be impaired by the menstrual cycle driven by the estradiol level. It has been shown that elevated estradiol level induces vaginal mucous secretion which in turn reduces antigen penetration of the vaginal epithelium, prevented CD8 T-cell priming and inhibited antigen uptake by APC across the vaginal epithelium following intravaginal immunization. This effect is reversed following the removal of the estradiol-induced mucous barrier with mucinases^117^. Indeed, this route has a potential in mRNA immunization for the control of HIV and other urogenital pathogens, either alone or in combination with other immunization routes.

## Considerations for mucosal delivery of mRNA vaccines

### The prospects

Virtually all the mRNA vaccines currently being developed against infectious diseases are designed for parenteral administration mainly *via* the subcutaneous or intramuscular routes. Notably, the licensed COVID-19 mRNA vaccines described above are also given parenterally. Although parenteral administrations of vaccines have been generally protective as they generate effective systemic immunity, they often induce a relatively weak local mucosal immunity which may be inadequate for the complete elimination of the pathogens from mucosal sites^6^. Furthermore, a parenterally immunized individual may develop a persistent infection on the mucosal surfaces, owing to inefficacious local mucosal immunity; this may subsequently be transmitted to unimmunized or immunocompromised individuals^118^. However, an effective mucosal vaccine is capable of inducing both potent mucosal and systemic immunity through a robust humoral immunity, mediated by sIgA (as discussed earlier), and cell-mediated immunity^33^. In addition, IgG is also produced at the mucosal surface following mucosal vaccination to play role in the neutralization of pathogens but the IgG concentration is relatively lower as they are rapidly degraded in the mucosal environment^6^. Mucosal vaccination also induces both long-term memory B and T cells via homing of effector and memory cells to the mucosal sites from the draining lymph node through the blood to the mucosal sites. As shown earlier, this is achieved through the homing receptors CCR9 and CCR10 expressed by the adaptive immune cells and are acquired in the lymph node^33^. The mucosal response to vaccines, like pathogenic stimulation, is largely driven by the presence of MALT at mucosal sites with inductive sites rich in abundance of APCs, B cells and T cells as described earlier. In addition, the specialized M cells at the epithelial surface actively trap mucosal vaccine antigens for delivery to the underlying immune cells of the MALT at the submucosal region^100^. No doubt, mRNA vaccines would benefit from these immunological advantages if administered *via* the mucosal route thereby fostering an intense protection at the mucosal portal of entry of the emerging viruses to limit infection and transmission.

### The challenges

The comparative advantages offered by mucosal vaccination in terms of broad range of immune protection spanning the mucosal and systemic immunity, including the non-immunological advantages offered in terms of patient’s compliance as well as the absence of local reactions form a very strong basis for the consideration of mucosal delivery of mRNA vaccines. However, mRNA vaccines, like other vaccines, are confronted with many challenges at the mucosal surfaces which must be overcome before exploiting the full potential of mucosal vaccination. For example, the tolerability issues remain a cogent reason why all the currently licensed mucosal vaccines are either live attenuated (*Vibrio cholerae*, *Influenza*, *Poliovirus*, *Salmonella typhimurium* and *Rotavirus* vaccines), whole-cell inactivated (*Vibrio cholerae vaccines*) or live re-assortant viruses (*Influenza*, *Rotavirus* and *Salmonella typhimurium* vaccines). These are efficient in breaking the tolerance through the induction of robust inflammatory response^95^. Also, at the respiratory mucosa, the mRNA vaccine, like other vaccines, faces challenges discussed earlier which are further compounded by mucosal RNAse degrative actions and epithelial barrier that impedes mRNA uptake^18^. In addition, the mRNA vaccine faces challenges relating to salivary dilution, epithelial barrier and immunotolerance at the sublingual mucosa while those of the vagina and rectal mucosa are due mainly to mucus and epithelial barriers as shown in the next figure. These hurdles are the main limiting factors against the exploitation of mRNA vaccines via the mucosal route; therefore, there is need to develop strategies to overcome these mucosal barriers thereby ensuring effective delivery and efficient uptake of the mRNA molecules by mucosal APCs to induce immune response in the MALTs.

### The existing currently licensed mucosal vaccines: lessons for mucosal mRNA vaccines

Although there have been several attempts to develop mucosal vaccines over the years, only a few of such vaccines have been licensed commercially for human use. Many vaccines that showed good results in animal models often failed in clinical trials. Owing to the difficulty of the mucosal microenvironment, most of the licensed vaccines are live attenuated to enable multiplication and induction of broad immunity. There are also licensed inactivated vaccines against *Vibrio cholerae* which are less immunogenic but relatively circumvent the safety issues associated with live vaccines^5,33^. Furthermore, most of the licensed vaccines are approved for oral use while only one is approved for use through the nasal route^33^. The live vaccines consist of attenuated bacteria or viruses that are made less virulent than the wild-type pathogen. These live attenuated vaccines have the dual advantage of intrinsic adjuvanticity and high antigenic availability. In addition, these vaccines can be in the form of vectorized live vaccines that consist of live viruses or bacteria that express the recombinant antigen from the pathogen of interest^33,95^, however, these vectored vaccines may be relatively less effective because of possible pre-existing vector immunity as seen with adenoviral vectors. The live vaccines may set up a local infection or be engineered to replicate in the mucosal environment to increase the antigenic load, an attribute that is relevant to overcome the mucosal tolerability^119,120^. One main challenge associated with the live vaccine is the balance between attenuation and immunogenicity. Attenuation could be achieved either by serial passages or molecular modification such as gene deletion. A trade-off exists between these two important properties such that an effectively attenuated vaccine is safe but less immunogenic and vice-versa^33^. A relatively safe and highly immunogenic vaccine is obtainable via molecular attenuation by precise gene mutation as seen with the case of *Salmonella* vaccine^121^. Another drawback of the live vaccines is the possibility for reversion to the virulent strain and the reactogenicity which pose a huge safety risk, especially in the immunocompromised, elderly or infants^93^. These safety issues are the main driving force for the consideration of non-living inactivated whole-cell or subunit mucosal vaccines. There are currently 3 licensed inactivated vaccines ((Dukoral, Shanchol and Euvichol) produced by inactivating the native pathogen with heat, chemical or radiation. Since these cannot multiply, they are generally safer than the live vaccines, however, they are less immunogenic and therefore require booster doses^5,33,93^. The subunit vaccines are generally much safer than the live or inactivated vaccines, but they are much less immunogenic, and their success have been limited due to the rapid degradation, poor uptake and the barriers in the mucosal environment. There is no licensed subunit mucosal vaccine, but the cholera toxin B (CTB) subunit is the only subunit antigen to be included in a licensed vaccine as an adjunct in the whole cell killed cholera vaccine (Dukoral) due to its binding affinity to receptors on the mucosal epithelial cells and its high immunogenicity^95,122,123^. The currently licensed mucosal vaccines in clinical use are summarized in Table 3 alongside their immunogenicities and efficacies.

#### Lessons

In the context of mRNA vaccine mucosal administration, some crucial lessons could be gathered from the perspective of licensed vaccines. First, it is imperative to note that most of the vaccines are live attenuated owing to the mucosal tolerance. Hence, it is worth considering when designing an mRNA vaccine formulation to have a construct that closely mimics a live pathogen which can multiply and allows persistent antigen presence. In this regard, a self-amplifying mRNA could be considered since the mRNA construct can multiply several times, owing to its inherent design as discussed earlier, although this requires the mRNA molecules to be delivered directly into the mucosal epithelial cells. This means that a delivery system is required to protect the sa-RNA molecules before uptake and intracellular delivery. For the conventional mRNA, a delivery system that closely mimics a live pathogen in terms of innate stimulation should be designed by including PRR ligands since it is practically impossible to have a multiplying delivery vehicle. Nonetheless, inactivated vaccines are also devoid of multiplicability and some mucosal vaccines with good efficacy are inactivated. Therefore, constructing the delivery system to include a microbial ligand as the case with the inclusion of CTB in Duchoral could help mimic inactivated vaccines and induce durable response. Overall, as shown in the table above, the vaccine-induced response with all the mucosal vaccines is driven by sIgA mucosal antibodies as well as systemic neutralizing IgG. For example, in the case of the licensed intranasal influenza vaccine, nasal IgA has been reported to be crucial for vaccine efficacy in children and this correlates with protection in human experimental challenge studies. In addition, T-cell response and innate immunity are important contributors to protective immunity in a similar way to immune response induced by the wild-type virus, a strong indication that site-specific immunization is key to have a response comparable to natural infection^44,124^. Furthermore, oral polio vaccine (OPV) containing a mixture of different live attenuated poliovirus serotypes has been reported to induce protection via both systemic and humoral immunity when administered at a complete dose. The systemic IgG response is effective in preventing neuroinvasion and avoid paralytic symptoms. The gastrointestinal IgA, which is unique to OPV compared to the injectable inactivated polio vaccine, is relevant in preventing person-person transmission since these antibodies control multiplication at the infection site (GIT)^5^. This is a strong indication that mucosal mRNA vaccine administration could indeed a robust response which would be strongly preventive against infectious colonization and/or systemic dissemination. Strategies to enhance the successful utilization of mucosal mRNA vaccines are suggestively discussed later in this review.

### Mucosal mRNA vaccine delivery vehicles: prospects from pre-existing experimental studies

In the context of mucosal exploitation, mRNA vaccines have been experimentally utilized mainly via the intranasal routes as well as intravaginal administration^125^, there is however no known oral mRNA vaccine administration till date,^118^ most probably due to the extensive challenges posed by the GI mucosal degradative environment. The nasal route is widely considered for mucosal delivery because it presents fewer challenges than the oral route due to the non-abundant secreted enzymes, lack of acidic environment, smaller surface area and lesser dose requirement compared to the oral route^42^. In addition, the nasal mucosa similarly possesses the nasal associated lymphoid tissue (NALT) which is rich in APC, B cells and T cells overlaid by M-cells which facilitate antigen capture and delivery^18,42^. There is a number of studies in which different delivery vehicles were explored with success in mucosal mRNA vaccine delivery. An early study in 2010 showed that intranasal administration of naked mRNA-Hsp65 which encoded the Hsp protein of *Mycobacterium leprae* induced protection against virulent *Mycobacterium tuberculosis* infection in an experimental mice 30 days after vaccination. There was robust production of Th1 cytokines namely interferon-gamma and TNF-alpha. The study further depicted the role of lung APC in capturing the mRNA molecules starting within 30 min and lasting up to 8 h post-vaccination^126^. However, in a more recent study, the role of an intranasal delivery system in driving a more robust response compared to naked mRNA was depicted. In this study, an increased nasal residence time of the mRNA molecules was achieved using a complex of cyclodextrin, a bioadhesive sugar molecule, coupled with low molecular weight polyethylenimine (PEI-2K) for the delivery vehicle of mRNA-gp120 encoding the gp120 antigen of HIV. Results from this study revealed that the CP2K-mRNA increased transfection efficiency in cell lines in-vitro. More importantly, intranasal administration in mice prolonged the residence time of mRNA in the nasal mucosa owing to the mucoadhesive property of cyclodextrin, and enhanced crossing of epithelial barrier by paracellular and transcellular transport with higher safety profile compared to high molecular weight PEI25k-mRNA system. In addition, CP2k-mRNA showed a more robust HIV gp120-specific cellular and humoral responses than naked mRNA or PEI25k-mRNA, this is depicted by comparatively higher systemic IgG and mucosal IgA at both nasal and vaginal mucosa, higher CD8+ and CD4+ cellular responses with a balanced TH1/TH2/TH17 cytokine responses^18^. In further studies, CP2k demonstrated an enhanced trafficking of mRNA molecules to the lymph node and triggered a more potent maturation of DCs in vivo after intranasal administration of CP2k-mRNA encoding OVA antigen when compared to CP600-mRNA (containing 600dalton PEI) and PEI25k-mRNA (without cyclodextrin)^127^. Sugar modification of LNP has been shown to enhance mucosal mRNA delivery. In a study on Influenza A H1N1 virus using cationic LNP delivery system, the protein expression in-vitro was relatively higher when the LNP was modified with mannose (LNP-man), a ligand of innate receptors, compared to the unmodified counterpart. Furthermore, protective humoral and cellular responses were reported following intranasal administration of 2 separate doses each of mRNA-H1HA-LNP and mRNA-H1HA-LNP-man encoding Influenza H1N1 HA protein. Although, both were effective in preventing weight loss and death in mice challenged with lethal dose of H1N1 virus two weeks post-immunization, the response was slightly higher in the LNP-man group signifying that ligand modification of delivery systems can drive a more potent response via the mucosal route^128^.

The intranasal route has also been explored in the delivery of mRNA encoding neutralizing antibodies against some infectious diseases. In one study, mRNA-encoded neutralizing antibodies achieved in vivo neutralization of RSV with minimal inflammation following aerosolized administration into the lungs. Furthermore, GPI-anchored mRNA-encoded neutralizing antibodies were retained on the plasma membrane of lung epithelial cells after expression and the neutralizing activity of the antibodies were effective against RSV with or without GPI anchor, a promising basis for mRNA-based pulmonary prophylaxis^129^. A similar result was reported in another research following intravaginal administration of aerosolized mRNA encoding neutralizing antibodies against HIV gp120, this similarly achieved expression of the anti-HIV gp120 neutralizing antibodies in the genital epithelium in sheep model^130^. There are few studies reporting intranasal administration of mRNA-based cancer vaccines leading to the regression of tumour growth. A research group reported that the intranasal administration of an mRNA-based cancer vaccine encoding chicken OVA (mRNA-OVA) delivered with a nanoparticle carrier showed potent prophylactic and therapeutic efficacy against tumour growth and progression in mice which were more potent than observed with naked mRNA-OVA or the negative control (mRNA-GFP). The result from this study also revealed robust OVA-specific CD8 T cells as the main driver of the tumour immunity and was present in the mRNA-OVA nanoparticle system^131^. A similar result was observed recently in another research following intranasal vaccination of mice with mRNA-CK19 vaccine encoding cytokeratin-19 which was encapsulated in a cationic liposome-protamine complex (LPC) delivery vehicle. A robust antitumor immunity was reported with abundant tumour-specific cellular responses and effective tumour size reduction in a therapeutic setting^132^. Overall, there are prospects from the experimental trials of mucosal delivery vehicles in favour of the mRNA vaccine. Of course, there exists a huge research gap in the field of mRNA mucosal vaccine delivery, hence, more extensive research is required to further unravel the potential of mRNA vaccines and to accelerate this success to clinical applications in mucosal vaccination.

### Innovative strategies to improve mucosal immunity

Efforts towards the improvement of mucosal vaccines have been focused on the development of novel delivery strategies which can overcome one or more of the challenges encountered at the mucosal environment with the sole aim of preserving antigen structural integrity, enhancing antigen bioavailability, and achieving successful induction of both mucosal and systemic immune responses. These strategies vary depending on the targeted inductive site and mucosal compartment^92,93^. The nature of the antigen formulation is a crucial determinant of the enhancing strategy to employ. Soluble vaccine antigens encounter impeded uptake by the mucosal immune cells due to the constantly renewing mucous barrier which limit antigen uptake. This issue can be overcome via the use of mucoadhesive molecules such as chitosan and starch which functions to allow a close contact between the antigen and the mucous membrane, thereby, increasing soluble antigen uptake after enhanced adhesion of the antigen to the mucosal surface. Chitosan has been shown to have a dual function of acting both as a mucoadhesive agent and as an adjuvant, hence it increases uptake and immunogenicity, perhaps sufficient to break the mucosal tolerance. In addition, it is possible to formulate an additional adjuvant together with chitosan with the aim of inducing robust immunity. Chitosan-based delivery systems have been explored with success for intranasal and intravaginal vaccination^33,133,134^. On the other hand, particulate vaccine antigens are generally more successful than the soluble antigens. This approach involves the encapsulation or intrinsic formulation of the antigens in form of particles which prevent rapid degradation of the antigens and can, at the same time, be manipulated for targeted delivery of antigen to APC and M-cells or to include adjuvants in the vaccine formulation. This includes the use of polymers, Virus-like particles, biodegradable microparticles such as Poly (lactic-co-glycolic acid) (PLGA) or nanoparticles such as liposome and bacterial ghosts. This strategy allows for a controlled and/or continued release of antigen while also preventing the degradation of the antigen and adjuvant before reaching the target site. It is also possible to include adhesive molecules such as lectin which offers the function of increasing residence time by bringing into contact the antigen and adjuvant with the mucosal surface. PLGA microspheres strategy allows 2 types of vaccine release, viz, controlled release whereby vaccine antigens are continuously released mimicking continuous boosters, and pulsatile release whereby vaccine antigens are released in two distinct time intervals mimicking 2-dose booster strategy^33,135^. The VLP is currently being explored as a mucosal delivery strategy. Novel techniques include the use of the VLP to co-deliver antigens together with an adjuvant. In a recent study, a chimeric VLP was bioengineered to express HA influneza antigen and coated with the protozoan *Giardia lamblia* surface protein, variant-specific surface protein (VSP). The VSP successfully protected the VLP system from degradative enzymes and PH fluctuations and provided an additional adjuvanting properties. Oral immunization of mice with this chimeric VSP-coated VLP expressing the influenza HA antigen induces a remarkable immune response and protected the mice following challenge with Influenza virus^136^. Lipid-based polymers represent another important strategy for particulate delivery of mucosal vaccines. The lipid-based polymers that have been explored include liposomes, bilosomes, virosomes and proteosomes and immune-stimulating complexes (ISCOMs). The proteosomes consist of *Neisseria* species-derived hydrophobic proteinaceous nanoparticles which are capable of associating with the hydrophobic domains of antigens and enhance delivery to the immune system. This strategy was explored in the intranasal delivery of a subunit influenza HA protein in mice and clinically in healthy human subjects where it induced nasal sIgA and systemic anti-HA antibodies^137^. Liposomes, as discussed earlier, are spherical nanovesicles consisting of a phospholipid bilayer and cholesterol possessing both hydrophobic and hydrophilic layers enclosing an aqueous inner core. This strategy offers an advantage of multiple antigen delivery simultaneously since different antigens localize at different portions of the liposome and can be used with different antigens including DNA and RNA platforms as introduced earlier^5,101^. Liposome-encapsulated DNA vaccine encoding the M1 protein of Influenza virus was reported to achieve an efficient in-vitro and in vivo expression of the encoded protein. Oral immunization of mice with this encapsulated DNA vaccine induces both cellular and humoral responses and augmented interferon-gamma response. Oral immunization of mice with this vaccine also protected from influenza challenge^138^. ISCOMs are colloidal structures synthesized from phospholipids, cholesterol and saponins. They play the dual role of acting as a carrier and as an immunostimulating agent for vaccine delivery. These ISCOMs are capable of entrapping different antigens and have been shown to deliver antigen to DCs and facilitate endosomal escape to induce T-cell and humoral responses. Intranasal delivery of an influenza split virus entrapped in ISCOM induced broad influenza-specific antibodies which protected against influenza challenge at a 10–100 fold lower dose than the split virus alone^139^. The bilosomes are similar to liposomes in structural organization and adjuvanticity but they are engineered to include bile salts in their formulation. The presence of bile salts offers a better advantage over liposome since liposomes can be disrupted by bile acid in the GIT while bilosomes remain stable and are unaffected by bile acid^5^. This strategy is being explored for oral vaccine delivery and have been shown to induce both sIgA humoral and cellular immunity and a balanced Th1/Th2 response following oral immunization with influenza subunit protein^140^. Cell-directed approaches are also explored to improve antigen uptake and vaccine response. This involves strategies targeted towards APC and M-cells on the mucosal surface since these cells are pivotal in driving mucosal and systemic responses. As stated in the previous sections, /M-cells are mucosal associated cells which can deliver mucosal antigens to DC or macrophages by transcytosis. Efforts are made to target antigen towards the M-cells by utilizing M cell ligands. For example, *Ulex europaeus* agglutinin 1 (UEA-1) which is a plant-derived lectin that specifically bind to M cell surface α-L-fucose residues, has been used as a strategy to target M cells. This has been used to modify the surface of a PLGA nanoparticle delivery system and was shown to induce systemic IgG and mucosal IgA in animal model after oral immunization^141^. Some bacterial proteins for which receptors are expressed on the M cells have also been employed in M cell targeting. For example, the GP2 receptor expressed on both murine and human M cell has been shown to bind FimH protein present on *E. coli* and *Salmonella typhirium*, in addition, outer membrane protein H found on Yersinia enterocolitica can bind a receptor on M cells. Therefore, recombinant vectors or delivery systems expressing these M cell ligands are being explored for M cells targeting^94^. A similar strategy is applied for mucosal DC targeting. Several DC receptors have been identified and are targeted for vaccine delivery, this includes DC-SIGN, CLEC, Langerin, DCIR, Dectin-1. Since DCs are widely distributed along the mucosal surface, including DC ligands in the antigen formulation would enhance vaccine uptake and drive vaccine response. Furthermore, some bacterial species such as *Lactobacillus acidophilus* have been engineered to express DC ligand and were shown to induce mucosal immunity that was protective against lethal *Bacillus anthracis* challenge following oral immunization^94,96,142,143^. As seen with other routes of administration, the immune response following mucosal immunization can be enhanced and facilitated using adjuvants. Mucosal adjuvants are more important since they target the broad mucosal epithelial cells rather than just the M cells or APCs that represent only a small fraction of the cells at the mucosal epithelia. The most utilized and best characterized mucosal adjuvants are the toxoids which are non-toxic derivatives of bacterial toxins originating from the enterotoxigenic *E. coli* (ETEC) *and Vibrio cholerae*, namely heat-labile toxin (LT) and cholera toxin (CT) respectively, both of which are ATP-ribosylating enterotoxins. These toxins have been modified such that they have lost their toxigenicity while still retaining their potent immunostimulatory properties^92^. Although these two adjuvants have potent immunostimulatory properties and are capable of breaking mucosal tolerance to induce mucosal and systemic responses, their residual toxicity issues have limited their clinical use^94^. In an attempt to reduce the toxicity of LT, mutation was introduced at 2 positions in the A subunit to generate a double mutant form (dmLT) which has been shown to be effective and well tolerated in preclinical and clinical studies^144,145^. Based on the same approach, CT has also been modified in a novel multiple mutated form (mmCT) which was shown to be non-toxic and induced a protective immunity with cellular and both mucosal and systemic antibodies^146^. Several other next-generation mucosal adjuvants are currently being explored based on the pattern recognition receptor (PRR) ligands since these ligands are widely expressed by different cells on the mucosal surfaces. Obviously, TLR agonists are mostly researched because of the wide distribution of TLRs across mucosal epithelial cells. Here, the most promising from preclinical evaluations are the TLR9 and TLR4 agonists such as unmethylated cytosine-guanine-containing oligonucleotides (CpG DNA) and monophosphoryl lipid A (MPL) respectively^147–149^. Other TLR agonists that have shown results include flagellin which is a TLR5 ligand, imiquimoid which is a TLR7/8 agonist, and poly I:C which is a ligand of TLR 3^96^. In addition to the TLR ligands, a promising adjuvant called α-GalCer which is a potent activator of the abundant mucosal invariant natural killer T cells (iNKT) has been shown in various preclinical studies to be a good strategy for enhancing mucosal immunity with protective cellular and humoral responses^150,151^. Cytokines have also been shown as a possible strategy to enhance mucosal immunity and improve the efficacy of mucosal vaccines. For example, IL-12 co-administration was reported to enhance vaccine response following intranasal immunization with HIV antigens^152^. More recently, intranasal administration of a recombinant IL-1beta with Influenza virus HA protein induced a protective immunity evidenced by both HA-specific and strain-specific neutralizing antibodies as well as the local activation of CD103^+^ CD69^+^ tissue-resident memory T cells (T_RM_)^153^. Recent studies have also reported the induction of mucosal immunity following parenteral immunization and this is mediated via the upregulation of gut-homing molecules by parenterally activated immune cells. This strategy requires the use of specific adjuvants. For example, it was shown that dmLT, but not CpG, enhanced the upregulation of the gut-homing α4β7 integrin by antigen-specific T cells following intradermal immunization and this was driven by CD103^+^ DCs^154^. Similarly, subcutaneous administration of retinoic acid with antigen induces the upregulation of α4β7 on both T and B cells leading to gut-homing of antigen-specific T cells and IgA^+^ B cells from the regional inguinal lymph node^155^.

## Conclusion and future possibilities

The results from the reviewed studies perhaps provide some basis for the promising potential for mucosal mRNA vaccines for the control of infectious diseases. The major challenge is the harsh mucosal environment and the epithelial barrier, there is a need to develop more efficient delivery systems which ensure the stability of the mRNA molecules and improve uptake across the epithelial barriers while also minimizing toxicity. The results from previous studies have shown the potential of polymer-based, lipid-based and hybrid delivery vehicles as reviewed above. The lack of experimental studies utilizing the oral mucosa is perhaps due to the more challenging nature of the GI environment in terms of varying pH and the presence of abundant degradative enzymes. There is a need to develop a delivery vehicle which can overcome these challenges. One possibility is by modifying the existing delivery systems which have shown success in experimental models. Future studies can focus on making these delivery vehicles safer and more effective, for example, rendering them more resistant to degradation in the mucosal environment or improving immunogenicity by the incorporation of adjuvants like PRR ligands into the delivery vehicles as seen with the case of mannose-modified LNP which has already been explored with mRNA delivery as discussed earlier. Other PRR ligands such as CpG oligonucleotide, flagellin and imiquimoid could also be explored in the mRNA delivery vehicle to break the oral tolerance. This also includes dmLT, mmCT and α-GalCer which have been previously experimented for mucosal application. These adjuvants could be expressed on the delivery vehicle or perhaps be alternatively mRNA-encoded and co-delivered with the antigen-encoding mRNA vaccine candidate. As discussed above, the problem associated with pH variation can be overcome engineering the delivery systems to include bile acids as seen with bileosomes which would probably render the carrier vehicle insensitive to the pH in the GIT. Another possibility is by increasing the dose especially for the oral route such that a high degree of the mRNA molecules is eventually taken up despite abundant degradative processes, this strategy however may not be economically advantageous and may be detrimental to vaccine coverage in a pandemic scenario. In the context of nasal and oral immunization strategies, engineering the mRNA delivery construct to target M cells at the mucosal surfaces would result in an increased effective uptake of the mRNA molecules to trigger robust immune response as discussed in the preceding section and this can be explored in future studies as well. Perhaps, the mRNA delivery vehicle could be designed to include M cell ligands such as UEA-1 or microbial ligand FimH which would preferentially enhance uptake by M cells. Another possibility is to use an antibody-guided targeting where the delivery vehicle is coupled to the Fc portion of an antibody whose Fab region is specific for an M cell surface molecule. One important challenge with mucosal vaccination particularly through the oral and sublingual routes is the tolerability potential as highlighted earlier; inclusion and co-delivery of cytokine or chemokine-encoding mRNA with the antigen-encoding mRNA may be a potential strategy of breaking this tolerance and triggering immunogenicity, in addition to PRR ligands. Since different cellular phenotype are required for different class of pathogens (e.g virus, bacteria and helminth), the specific cytokines that imprint the desired T cell response could possibly be mRNA-encoded and co-delivered with the antigen-encoding mRNA. For example, including IFN-γ for TH1/CD8 response or IL-17 for TH17 response against viruses and extracellular bacteria/fungi respectively. This strategy can be combined with a cell-targeted delivery to induce a significant response that breaks tolerance, but care should be taken to avoid detrimental inflammatory response. Since DCs are distributed all over the mucosal compartments, targeting these cells is indeed a promising strategy for vaginal and rectal mucosa which are devoid of M cells or organised lymphoid tissues and of course, other mucosal compartments. Numerous DC ligands have been employed in the context of DC targeting such as DC-SIGN, DCIR and CLEC-9a, these can be included in the delivery vehicle of mRNA vaccines intended for the sublingual, vaginal or rectal immunization as does with the oral and nasal routes. Another possibility is to couple the LNP or any other delivery vehicle to the Fc portion of an antibody whose Fab region is specific for a DC surface molecule. Indeed, DC targeting could potentially limit off-target mRNA vaccine uptake and can be used to preferentially target a specific DC lineage which would shape the T cell response towards the desired helper phenotype. The mucosal compartmentalization has been shown to impact the tolerogenic potential at different segments of the GIT. The upper small intestinal segment has been shown to have a higher tolerogenic propensity than the distal GI segments^156^. Hence, developing a delivery vehicle that bypasses the upper intestine may be an important strategy. Mucoadhesive substances which increase the residence time of the mRNA molecules have been proven with success in experimental models as seen above with cyclodextrin^18,127^ and other mucoadhesive substances used for vaccine platforms other than mRNA vaccines such as chitosan and starch^157–160^. Research can be focused on incorporating these substances in the delivery system with particular emphasis on toxicity avoidance. This would increase residence time, a property that is very relevant for nasal and sublingual immunization to overcome mucociliary action and salivary dilution respectively. Since the mucous layer can serve as a barrier to the delivery of substances across mucosal surfaces, perhaps engineering the delivery system to ensure efficient mucus-penetrating activity may improve antigenic mRNA delivery and this can be explored in future studies. Since it has been shown that gut-homing integrins and retinoic acid could induce homing of DC, B cells and T cells to the mucosal surface following parenteral immunization, it could be possible to direct efforts towards the inclusion of these molecules in the mRNA carrier molecule or encode these in the mRNA formulation and use this as a combination strategy to mucosal mRNA immunization. Finally, it would be of immense benefit if mRNA could be engineered and constructed to be resistant by virtue of its inherent design such that it becomes RNAse insensitive and able to withstand the harsh mucosal environment. This requires extensive research not only regarding the design and delivery but also to monitor the safety of the mRNA vaccines administered mucosally. Although most of the strategies we proposed have been explored for other vaccine platforms, as it stands now, most of these strategies are rather speculative in the context of mRNA vaccine delivery. Nonetheless, we believe that research efforts to exploit the proposed strategies would accelerate and facilitate effective utilization of mucosal delivery to attain the full potential of mRNA vaccine in the fight against infectious diseases.

### Reporting summary

Further information on research design is available in the Nature Research Reporting Summary linked to this article.

## Supplementary information

REPORTING SUMMARY
